# Heat fixation inactivates viral and bacterial pathogens and is compatible with downstream MALDI mass spectrometry tissue imaging

**DOI:** 10.1186/s12866-015-0431-7

**Published:** 2015-05-13

**Authors:** Lisa H Cazares, Sean A Van Tongeren, Julie Costantino, Tara Kenny, Nicole L Garza, Ginger Donnelly, Douglas Lane, Rekha G Panchal, Sina Bavari

**Affiliations:** Henry M. Jackson Foundation, Maryland, USA; DoD Biotechnology High Performance Computing Software Applications Institute (BHSAI/TATRC), Frederick, Maryland USA; Division of Molecular and Translational Sciences, US Army Medical Research Institute for Infectious Diseases (USAMRIID) Fort Detrick, Maryland, USA

**Keywords:** Tissue fixation, Proteomics, Microbial inactivation, Matrix assisted laser desorption ionization mass spectrometry (MALDI-MS), Tissue imaging

## Abstract

**Background:**

Tissue samples should be fixed and permanently stabilized as soon as possible ex-vivo to avoid variations in proteomic content. Tissues collected from studies involving infectious microorganisms, must face the additional challenge of pathogen inactivation before downstream proteomic analysis can be safely performed. Heat fixation using the Denator Stabilizor System (Gothenburg, Sweden) utilizes conductive heating, under a mild vacuum, to rapidly eliminate enzymatic degradation in tissue samples. Although many studies have reported on the ability of this method to stop proteolytic degradation and other sample changes immediately and permanently, pathogen inactivation has not been studied.

**Results:**

We examined the ability of the heat fixation workflow to inactivate bacterial and viral pathogens and the suitability of this tissue for Matrix Assisted Laser Desorption Ionization mass spectrometry imaging (MALDI-MSI). Mice were infected with viral or bacterial pathogens representing two strains of Venezuelan Equine Encephalitis virus (VEEV) and two strains of *Burkholderia*. Additionally, a tissue mimetic model was employed using *Escherichia, Klebsiella* and *Acinetobacter* isolates. Infected tissue samples harvested from each animal or mimetic model were sectioned in half. One half was heat fixed and the other remained untreated. Lysates from each sample were checked for organism viability by performing plaque (infectivity) assays or plating on nutrient agar for colony forming unit (CFU) calculation. Untreated infected control tissue demonstrated the presence of each viable pathogen by positive plaque or colony formation, whereas heat fixation resulted in complete inactivation of both the viral and bacterial pathogens. MALDI-MSI images produced from heat fixed tissue were reflective of molecular distributions within brain, spleen and lung tissue structures.

**Conclusions:**

We conclude that heat fixation inactivates viral and bacterial pathogens and is compatible with proteomic analysis by MALDI-MSI. This treatment will enable the use of infected tissue from studies performed in bio-safety level 3 laboratories with VEEV and *Burkholderia* to be safely used for proteomic, small molecule drug detection, and imaging mass spectrometry analysis.

## Background

Many challenges must be overcome for successful proteomic analysis in tissue samples. When sampling is performed in a manner optimal for experimental reproducibility and is a representative “snapshot” of the organisms living state, the highest sample quality for experimental analysis is achieved. Endogenous proteolytic activity begins immediately following disruption of oxygen and nutrients, and can continue during storage and sample processing [1]. Therefore, in order to obtain reliable and consistent results, tissue samples should be fixed and permanently stabilized as soon as possible ex-vivo to avoid significant variations in proteomic content throughout the experimental processes [2,3]. Tissue samples collected from animal studies involving infectious microorganisms must face an additional challenge of pathogen inactivation before downstream proteomic analysis can be safely performed in a routine laboratory environment (biosafety level 2). Therefore, biosampling procedures for infectious disease research must be carefully considered and optimized for both experimental integrity and safety.

Fixation of tissues can be accomplished by chemical or physical treatment. Physical methods include cryo-preservation, heating, and micro-waving. The most common chemical fixation method is 10% neutral buffered formalin. This method is ideal for preserving cellular morphology and inactivates most pathogens, but produces extensive covalent crosslinking within proteins creating a challenge for subsequent proteomic and mass spectrometric analysis. These crosslinks can be partially reversed using antigen retrieval methods, to achieve maximum detection of proteins and peptides [4]. However, amino acids, carbohydrates, lipids, phosphates, proteins and ions, such as Cl(−) and K(+), have been shown to leach from tissue sections into the aqueous fixative medium, further creating sources of variability in molecular content [5]. Alcohol based fixatives can also preserve morphology and are more compatible with proteomic analysis [6], but suffer from the same potential problem of analyte leaching.

The most common physical method of tissue stabilization used in proteomic and mass spectrometry analysis is snap-freezing and cryo-preservation. Although this method can be performed very quickly after tissue harvest, enzymatic activity and degradation will begin again once the sample is thawed for processing or sectioning [7,8] and pathogens present in the tissue remain viable. Heat or microwave-irradiation-enhanced fixation was first demonstrated in 1970 [9] and can be performed to enhance and shorten the times for chemical fixation or be used alone. Recently a method of heat fixation utilizing a combination of heat and mild vacuum was reported to be a superior method for proteome stabilization in tissue when compared to snap-freezing [10]. Heat stabilization using the commercially available Denator Stabilizor System is compatible with major proteomic workflows and is beneficial in preserving peptides, proteins and their modifications in tissue samples [11-13]. In addition, heat stabilized tissues exhibit similar intensity distribution for marker ions as that seen in frozen tissue sections used for matrix assisted laser desorption ionization MALDI-MSI [8]. Although many studies have reported on the ability of this method to stop proteolytic degradation and other sample changes immediately and permanently, pathogen inactivation has not been studied.

MALDI-MSI is a powerful tool for the generation of multidimensional spatial expression maps of biomolecules directly from a thin tissue section. Cells, tissue sections or even whole body slices from small animals are deposited onto a conductive glass or metal plate and covered with matrix (usually a UV-absorbing weak organic acid dissolved in a volatile solvent). The analytes in the tissue are extracted by the solvent and co-crystallize with the matrix compound, which then facilitates the transfer of energy of the MALDI laser allowing for the desorption/ionization of the analyte molecules. Mass spectra can then be acquired across the tissue at defined geometrical coordinates resulting in ion intensity maps or images depicting the relative level of each detected analyte. MALDI ion sources are well suited to this application since they enable the ionization of diverse biomolecules, including peptides, proteins, oligonucleotides, sugars, lipids, and small molecule drugs when the appropriate matrix is employed [14-16].

Unlike traditional proteomic techniques where spatial information is lost due to extraction and purification, MALDI-MSI allows for the simultaneous detection of many analytes while maintaining their spatial distribution within a histologically defined tissue section. MALDI-MSI is generally a non-targeted approach and does not require any labels or specific probes. This makes MALDI-MSI an extremely useful biomarker discovery tool as it facilitates the collection of multiplexed data on the specific localization of molecular ions in tissue sections, and allows for the measurement and mapping of variations of these ions during development and disease progression or treatment. Therefore MALDI-MSI is valuable tool for monitoring the response to infection with highly pathogenic organisms for the discovery of biomarkers and therapeutic targets. This work describes the effectiveness of heat fixation using the Denator instrument for the inactivation of bacteria and viruses and the compatibility of this treatment with MALDI mass spectrometry imaging.

## Results

### Experimental design

**Figure 1:**
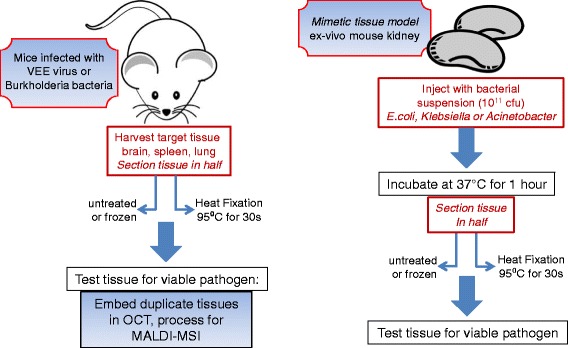
Workflows of experimental design to determine the ability of heat fixation to inactive viral and bacterial pathogens.

The workflows used to evaluate the ability of heat fixation using the Denator Stabilizor T1 system to inactivate viral and bacterial pathogens in tissue samples are shown in Figure 1. Our experimental design included both an in-vivo infection model as well as a tissue mimetic model. Infected tissue samples were sectioned in half and heat fixed or left untreated, followed by pathogen viability assays. We chose to begin our investigation using a “custom” heat fixation method of 95°C for 30 seconds in the T1 Stabilizor instrument with the upper heater position at 90%. The temperature, heater position and stabilization time are settings that can be adjusted when selecting the “custom” method option. This custom setting is based on the default fresh structure preserving method of 90-95°C for 10–20 seconds for most tissues.

The structure preserving method evacuates the stabilizer card of air before heating takes place (to reduce the risk of oxidation), but does not compress the tissue with further vacuum. The time and temperature were chosen as starting points based on basic heat- inactivation data of bacteria and viruses in foods [17-19]. According to the manufacturer’s guidelines, the T1 Stabilizor instrument is compatible with tissue sample sizes up to 33 mm in diameter and 7 mm in height, and there is no lower limit for sample size. To determine the exact amount of energy required for complete heat fixation, the instrument uses algorithms which combine the details of the physical state (fresh or frozen) and the sample size (measured automatically by lasers).

### Inactivation of viral pathogens in infected tissues

To evaluate if the heat fixation protocol inactivates viral pathogens from infected tissues, Venezuelan equine encephalitis virus (VEEV), an enveloped single stranded positive RNA virus classified in the Alphavirus genus and Togaviridae family was selected for testing. VEEV infection in mice begins with the lymphoid tissues, followed by viremia and penetration into and infection of the central nervous system [13]. In this study, BALB/c or C57BL/6 mice were infected (i.n. or s.c) with the vaccine or virulent strain of VEEV. Spleen and brain tissues harvested from infected mice were left untreated, or subjected to heat fixation and then lysates were prepared to determine viremia. As shown in Table 1, the presence of viable viral pathogen VEEV, (TC83 vaccine strain and virulent Trinidad donkey strain) was confirmed in the untreated mouse brain tissue lysates by positive plaque assays on Vero-76 cells. Viral titers from the TC83 VEEV infected untreated brain tissue lysates (100-120 mg) ranged from 2.5 × 10^4^ to 5.5 × 10^4^ pfu/ml. Higher titers were obtained from untreated brain tissue harvested from mice infected with the virulent VEEV strain, ranging from 9.9 × 10^7^ to 4.2 × 10^8^. Lysates derived from the untreated spleen of mice infected with the virulent strain of VEEV exhibited titers of 6.5 × 10^3^-2.5 × 10^6^, however, no virus was recovered from the spleen tissue lysates derived from the TC83 infected mice. This is not surprising since mice challenged with live attenuated vaccine strain TC83, develop lower virus titers in the blood and brain and do not exhibit the histo-pathological lesions that occur in the spleen, lymph nodes, and brain tissue of mice infected with the virulent strain [20]. Most importantly, the heat fixed brain and spleen tissue samples produced no positive plaques indicating that both strains of VEEV were completely inactivated by the treatment.

**Table 1:** **Viable virus determination from untreated and heat fixed VEEV (vaccine strain and virulent strain) infected tissue**

**Pathogen**	**Tissue**	**Weight (mg)**	**Treatment**	**Dilution and plaque count**						**Average titer (pfu/ml)**
				**10** ^**−1**^	**10** ^**−2**^	**10** ^**−3**^	**10** ^**−4**^	**10** ^**−5**^	**10** ^**−6**^	
*VEE* (TC83) vaccine strain	brain	100	none	100+	21	3	0	0	0	2.5E + 04
		120	none	100+	30	8	0	0	0	5.5E + 04
		140	HF	0	0	0	0	0	0	0.0E + 00
		125	HF	0	0	0	0	0	0	0.0E + 00
	spleen	47	none	0	0	0	0	0	0	0.0E + 00
		52	none	0	0	0	0	0	0	0.0E + 00
		55	HF	0	0	0	0	0	0	0.0E + 00
		50	HF	0	0	0	0	0	0	0.0E + 00
*VEE* (Trinidad donkey) virulent strain	brain	210	none	TNTC	TNTC	TNTC	TNTC	TNTC	42	4.2E + 08
		240	none	TNTC	TNTC	TNTC	TNTC	58	14	9.9E + 07
		220	none	TNTC	TNTC	TNTC	TNTC	TNTC	37	3.7E + 08
		190	none	TNTC	TNTC	TNTC	TNTC	TNTC	34	3.4E + 08
		190	HF	0	0	0	0	0	0	0.0E + 00
		180	HF	0	0	0	0	0	0	0.0E + 00
		260	HF	0	0	0	0	0	0	0.0E + 00
		210	HF	0	0	0	0	0	0	0.0E + 00
	spleen	60	none	TNTC	TNTC	42	7	0	0	2.5E + 06
		50	none	45	13	0	0	0	0	8.8E + 03
		50	none	39	11	0	0	0	0	7.5E + 03
		70	none	38	9	0	0	0	0	6.5E + 03
		50	HF	0	0	0	0	0	0	0.0E + 00
		60	HF	0	0	0	0	0	0	0.0E + 00
		50	HF	0	0	0	0	0	0	0.0E + 00
		70	HF	0	0	0	0	0	0	0.0E + 00

TNTC = too numerous to count, HF = Heat fixed.

### Inactivation of bacterial pathogens in infected tissues

To evaluate the ability of the heat fixation method to inactivate bacterial pathogens, the Gram negative bacilli *Burkholderia thailandensis*, an avirulent strain, and *Burkholderia mallei,* a virulent strain that causes glanders disease, were selected for study. The initial stage of infection in BALB/C mice with both of these strains results in the localization of the pathogen in the upper and lower sections of the respiratory tract and transportation of bacteria within alveolar macrophages to regional lymph nodes [21]. Mice were infected via the i.n. route with *B. thailandensis* or *B. mallei* and after 4 days, spleen and lung tissues were harvested and subjected to either no treatment or heat fixation. Tissues lysates were prepared for a bacterial viability assay. As shown in Table 2, lysates from the untreated lung tissue harvested from mice infected with *B. thailandensis* or *B. mallei* exhibited colony counts in excess of 10^5^ CFU/ml, indicating that both bacteria invaded the lungs of all infected mice. Viable bacterial counts in the untreated spleen of *B. thailandensis* infected mice ranged from 7.0-7.6 × 10^4^ CFU/ml. Conversely, there were no viable bacteria detected in heat-fixed lung or spleen tissue, indicating complete inactivation. Spleen tissue was not harvested from the *B. mallei* infected mice due to the fact that bacterial numbers are consistently higher in the lungs than in other tissues when an aerosol or intranasal infection method is used [21].

**Table 2:** **Viable bacteria determination from untreated and heat-fixed** ***Burkholderia*** **infected tissue**

**Pathogen**	**Tissue**	**Weight (mg)**	**Treatment**	**cfu for**	**Average cfu/ml**
				**10** ^**−1**^	
*B. thailandensis*	lung	40	none	1000+	>1 × 10^5^
		52	none	1000+	>1 × 10^5^
		40	none	1000+	>1 × 10^5^
		56	HF	0	0.00
		48	HF	0	0.00
		34	HF	0	0.00
	spleen	61	none	70	7.0 × 10^4^
		64	none	71	7.1 × 10^4^
		71	none	76	7.6 × 10^4^
		57	HF	0	0.00
		45	HF	0	0.00
		65	HF	0	0.00
*B. mallei*	lung	ND	none	1000+	>1 × 10^5^
		ND	none	1000+	>1 × 10^5^
		ND	none	1000+	>1 × 10^5^
		ND	HF	0	0.00
		ND	HF	0	0.00
		ND	HF	0	0.00

HF = Heat fixed, ND=not determined.

In an effort to limit the use of animals for this study, we employed a tissue mimetic model to further investigate the ability of heat fixation to inactivate Gram negative bacteria from 3 families (*Enterobacteriaceae, Moraxellaceae, and Burkholderiaceae*), and 4 different genera: *Escherichia coli* (3 strains), *Klebsiella pneumoniae, Acinetobacter baumannii* and *Burkholderia thailandensis*. Whole kidneys harvested from uninfected control animals were inoculated with the bacterial isolates (one isolate for each kidney) and incubated at 37°C for 1 hour. After incubation, the kidneys were sectioned in half and one half was heat fixed and the other half was left untreated. Lysates of each tissue were made as previously described and viable bacterial counts were determined by plating serial dilutions on Lysogeny broth (LB) agar. As shown in Table 3, viable bacterial colonies were detected for each isolate tested in all the untreated kidney tissue (>10^3^ CFU/ml). No viable bacteria was detected in the kidney tissue which was heat fixed at 95°C for 30 seconds, indicating complete inactivation of all bacterial isolates present in the tissue mimetic.

**Table 3:** **Bacterial viability determination from untreated and heat-fixed kidney tissue obtained from a tissue mimetic model**

**Bacterial isolate**	**Treatment**	**1:10**	**1:100**	**1:1000**	**cfu/ml**
*E.coli* Top10	none	TNTC	1000+	1000+	>10^3^
*E.coli* pGSV3-GmR	none	TNTC	1000+	1000+	>10^3^
*E.coli* pAM17-KanR	none	TNTC	1000+	1000+	>10^3^
*Klebsiella pneumoniae*	none	TNTC	1000+	1000+	>10^3^
*Burkholderia thailandiensis*	none	TNTC	1000+	1000+	>10^3^
*Acinetobacter baumani*	none	TNTC	1000+	1000+	>10^3^
Control (no bacteria)	none	0	0	0	0
*E.coli* Top10	heat fixed	0	0	0	0
*E.coli* pGSV3-GmR	heat fixed	0	0	0	0
*E.coli* pAM17-KanR	heat fixed	0	0	0	0
*Klebsiella pneumoniae*	heat fixed	0	0	0	0
*Burkholderia thailandiensis*	heat fixed	0	0	0	0
*Acinetobacter baumani*	heat fixed	0	0	0	0
Control (no bacteria)	heat fixed	0	0	0	0

TNTC = too numerous to count, HF = Heat fixed.

### Evaluation of heat fixed tissue for compatibility with MALDI-MSI

**Figure 2:**
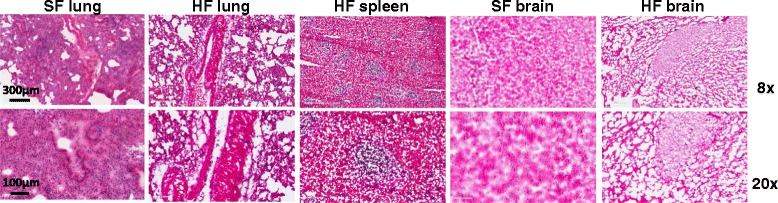
H&E stained sections of snap frozen (SF) and heat fixed (HF) mouse tissue showing morphological detail at 8x (top panels) and 20x (bottom panels) magnification. Tissues were heat-fixed at 95°C for 30 seconds or untreated and prepared for sectioning by embedding in OCT.

To evaluate the tissues for gross morphology and MALDI-MSI, duplicate samples were heat-fixed and prepared for sectioning by embedding in OCT and freezing at −80°C. One untreated brain tissue and one untreated lung tissue were also embedded in OCT. Cryo-sectioning was performed and 10 μm sections were placed on ITO coated slides for MALDI-MSI. Serial sections were H&E stained to evaluate tissue morphology after heat fixation (95°C for 30 s). Upon gross examination, heat fixation increased the rigidity of the tissue samples and the tissue appeared darker and slightly reduced in overall size. Additionally, the tissue was more prone to fracturing during cryo-sectioning as compared to snap-frozen tissue. As seen in Figure 2, the overall tissue structures are intact, but morphology changes were observed at the cellular level. Heat-fixed tissue displayed morphological changes similar to those observed in most snap-frozen sections. The expansion of water in the tissue upon freezing causes a consistent distortion, creating a “swiss cheese” effect. Some of these holes may be attributed to ice crystal formation, although the snap-frozen brain, embedded using the same method of immersion in pre-chilled isopentane bath cooled with dry ice, did not display these holes. It has been proposed that these small holes may be caused by the formation of steam pockets within the tissue during the rapid heating process [8].

**Figure 3:**
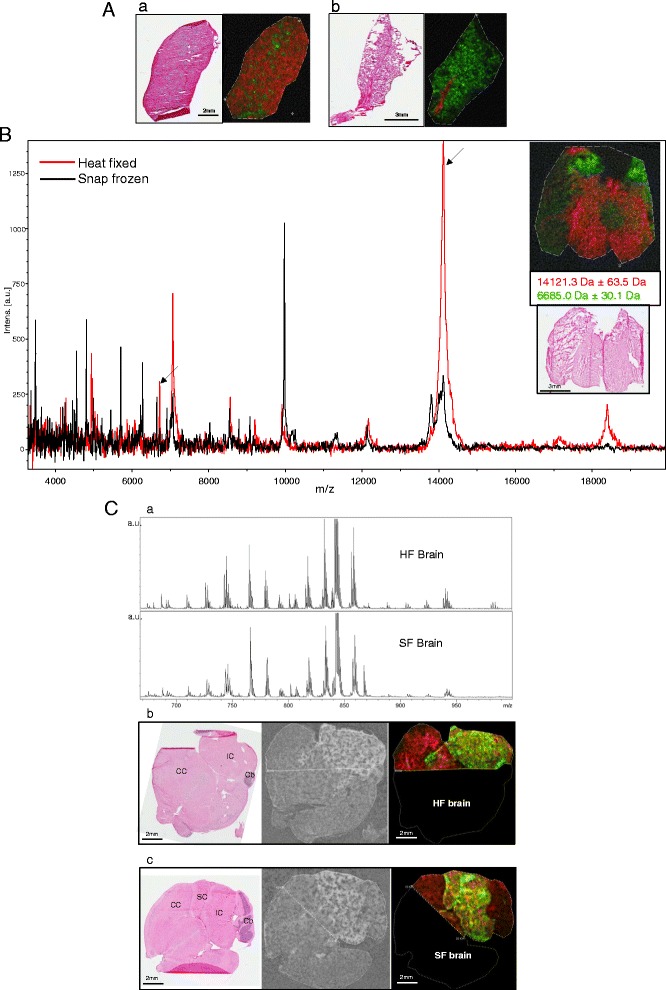
**A**. MALDI-MSI of heat fixed tissue after pathogen inactivation. Tissue sections (10 µm) were prepared for MALDI-MSI of proteins and peptides by spraying with sinapinic acid. a) H&E stained spleen serial section and pseudocolored MALDI-MSI ion overlay showing the expression pattern of *m/z* 8416 (green: located in areas of periarteriolar lymphocyte sheath), and *m/z* 14986 (red: surrounding tissue). b) H&E stained serial lung tissue section and pseudocolored image overlay showing the expression pattern of *m/z* 12167 (red: located in the primary bronchus), and *m/z* 13800 (green: surrounding tissue). All masses are reported with a mass window of 0.4%. **B**. Overlay of representative MALDI-MSI spectra (500 combined laser shots) obtained from corresponding regions of heat fixed (red trace) and snap frozen (black trace) mouse brain tissue. Arrows indicate the ions mapped in the inset image. Inset- pseudocolored ion image (top) and H&E of an adjacent section (bottom) of a heat fixed mouse brain section showing the distribution of *m/z* 14121 (red) and *m/z* 6688 (green) located in two regions of the midbrain. **C**. MALDI-MSI of heat fixed and snap frozen mouse brain tissue for lipid detection. (a) Average total MALDI spectra in reflector negative ionization mode obtained from heat fixed (HF) and snap frozen (SF) mouse brain display numerous peaks representing lipid ions in the *m/z* 700-950 region. (b-c) Scanned images of H&E stained and matrix (DHB) sprayed serial sections (left 2 panels) and pseudocolored overlays of ion images (right panels) in heat fixed (HF) (b) and snap frozen (SF) (c) mouse brain. Horizontal mouse brain sections showing the expression pattern of two lipid species: *m/z* 885.6 (green) in the inferior and superior colliculus and *m/z* 715.5 (red) in the cerebral cortex. Dashed lines indicate MALDI-MSI measurement regions. All masses are reported with a mass window of 0.1%. IC- inferior colliculus, SC-superior colliculus, CC- cerebral cortex, Cb-cerebellum.

The preservation of distinct morphological structures in the tissue is apparent in the MALDI-MSI images produced from heat fixed brain, spleen and lung tissue. As seen in Figure 3A, heat fixed spleen tissue exhibited differential expression of a peak at *m/z* 8426 ± 46 localized to the areas of periarteriolar lymphocyte sheath (PALS), and heat fixed lung tissue displayed a peak at *m/z* 12167 ± 67 unique to the primary bronchus. The large holes seen in the serial H&E sections are not visible in the MALDI-MSI images of the lung and spleen. This is most likely due to analyte diffusion into these areas during tissue washing or matrix application. Brain tissue structure is also preserved and displays ions with differential expression in 2 regions of the midbrain (Figure 3B inset). This is an indication that the changes to the fine structure observed in the H&E of the heat fixed tissue did not affect our ability to detect differential expression of peptides and proteins by MALDI-MSI, producing images reflective of the underlying morphology. In addition, as shown in Figure 3B, the spectral profile of the heat fixed brain tissue exhibits increased intensity for peaks above 12 kDa as compared to frozen spectra from the same brain region. Conversely spectra from untreated tissue displayed a larger number of low molecular weight ions, consistent with protein degradation.

In order to evaluate the effects of heat fixation with pathogen inactivation on smaller tissue analytes, MALDI tissue imaging for lipids was performed on snap frozen and heat fixed brain sections. For this analysis an effort was made to section and image similar brain regions for better comparison of brain structures and the molecules detected. Horizontal sections of mouse brain where made through regions of the cerebrum and cerebellum, sprayed with DHB matrix and subjected to MALDI-MSI in reflector negative ion mode. As shown in Figure 3C, the average total spectra exported from the compiled MALDI-MSI data set for heat fixed and snap frozen mouse brain both display numerous peaks representing lipid ions in the m/z 700–950 region. Based on previous published analysis of lipids mapped to mouse brain, the major classes of lipids visible in heat fixed and snap frozen brain tissue are glycerophospholipids such as phosphatidylinositol (PI), phosphatidylethanolamine (PE) and phosphatidylserine (PS), and phosphosphingolipids such as sphingomyelin [22]. These lipid classes are frequently observed in brain tissue via MALDI-MS in negative ion mode [23,24]. MALDI-MSI ion images produced for two ions at m/z 715.5 and m/z 885.6 show differential expression within different brain regions, but the pattern of expression is almost identical in the heat fixed and frozen brain sections. The ion at m/z 885.6 is present only in the inferior and superior colliculus, and corresponds well to the deprotonated form of phosphoinositol (38:4) which has a mass of 886.56 and is frequently observed in MALDI-MSI lipid analysis of mouse and rat brain tissue [23]. The ion at m/z 715.5 is primarily located in the cerebral cortex and has a similar mass to deprotonated PE-Cer/Sphingomyelin (C35:1 m/z 716.58). The similarity of the acquired spectra and images produced from heat fixed and frozen brain tissue indicates that the lipid profile is preserved after the pathogen inactivation/heat fixation process.

## Discussion

Proteomic analysis of tissue infected with bacterial or viral pathogens poses a dual challenge since both microbial inactivation and tissue stabilization must be considered. The use of fresh or frozen tissue samples is not an option for studies involving BSL-3 or BSL-4 level pathogens. Although formalin fixation of such tissues is performed, the prolonged fixation protocols (14–30 days) often dictated to ensure pathogen inactivation, render tissue virtually unusable for proteomic analysis due to extensive protein crosslinking. Heat-fixation using the Denator Stabilizor T1 instrument has been successfully applied to many proteomic workflows using tissue, including 2D electrophoresis [25], analysis of protein phosphorylation and other post-translational modifcations [11,26], but the use of heat fixation for microbial inactivation has not been studied previously. Recent reports by Goodwin et al. [8,27,28] have determined that heat fixation, using the basic structure preserving protocol, is compatible with MALDI-MSI, and produces protein profiles and ion images similar to, but more stable over time than fresh frozen. Since our method of pathogen inactivation requires a longer heat-fixation time, we sought to ensure that this treatment did not impair tissue morphology and structure to a degree that would preclude its use for MALDI-MSI.

Our data indicate that heat fixation at 95°C for 30 seconds is adequate for the inactivation of a + ssRNA enveloped virus in infected tissue samples. Initially, we chose as our guideline the temperatures and times used to inactivate viruses and bacteria in animal meat (pork beef or chicken) or shellfish [18,19,29,30]. Complete inactivation of the Hepatitis A virus, (a non-enveloped + ssRNA virus) in shellfish can be achieved by heat treatment at 90°C for 1.5 minutes [18]. In pilot experiments, we chose time points of 30 sec.-1.5 min. at 95°C for heat fixation of VEEV (TC83) infected tissue (data not shown). However, since VEEV was successfully inactivated at 30s, we did not perform the longer fixation times in subsequent studies.

It is often assumed that small non-enveloped viruses are among the most stable viruses and that viral sensitivity to heat increases with size and the presence of a viral envelope. However, a recent study measuring the thermal stability of many different viruses in a variety of matrices, did not find a relation between viral structure and thermal stability [31]. This study reported that most viruses were inactivated by heating to 73°C for 3 minutes in a liquid matrix and that virus infectivity loss occurs much more rapidly than does loss of amplifiable viral RNA or DNA. Indeed, Baert et al. reported that RT-PCR determination for the presence of mouse norovirus (MNV) did not correlate with the infectivity of virus particles after heat treatment [32]. Therefore, the loss of virus infectivity observed after heat treatment is most likely due to denaturation of the capsid protein. Further studies are necessary to determine the heat fixation parameters required for the inactivation of viruses from non-enveloped and “naked” virus types.

The method of heat fixation using the Denator T1 Stabilizor was adequate for the inactivation of Gram negative bacteria from 3 families (*Enterobacteriaceae, Moraxellaceae*, and *Burkholderiaceae*). It is likely that this treatment will inactivate most other gram negative bacterial isolates. Since the cell wall of Gram-negative organisms is much more complex than the cell wall of Gram-positive bacteria, our heat fixation parameters may be adequate for inactivation, with the exception of spores. Further studies using tissue infected with Gram positive bacteria are necessary to ensure safe handling after heat fixation.

Our MALDI-MSI images are reflective of molecular abundances and distributions within tissue structures in heat fixed brain, spleen and lung tissue. By embedding heat fixed tissue in OCT, cryo-sections can be obtained although the tissue is prone to fracturing. To alleviate this problem, fragile heat-fixed tissue has been cryo-sectioned with the assistance of carbon conductive tape, in preparation for MALDI-MSI [27]. The tissue morphology we observed after heat fixation is of adequate integrity to obtain MALDI-MSI images and spectra of good quality, however, changes to cellular fine structure were observed. The source of these tissue changes and/or artifacts has not been determined. The protein/peptide spectra produced from heat-fixed brain tissue displays many common ions with spectra from fresh-frozen tissue, although there were fewer low molecular weight ions visible in the average spectra exported from heat fixed brain tissue. Since a direct comparison of heat-fixed to frozen tissue profiles was not the goal of this study, an in-depth analysis of the spectral profiles was not performed. However, this observation is consistent with a recent study by Stingl et al., which reported fewer ex-vivo formed peptides in rodent brain tissue heat-fixed samples as compared to fresh frozen material [3].

MALDI-MSI has been widely used to analyze a variety of lipids in tissue sections [33-36]. Due to the high content and diversity of lipids in the central nervous system, brain tissue often serves as a model for MALDI-MSI method development. We therefore sought to determine the impact heat fixation had on lipid detection within mouse brain sections using MALDI-MSI. Brain tissue heat fixed for pathogen inactivation produced a pattern of lipid expression similar to snap frozen brain tissue indicating that the treatment did not change the lipid analyte content or distribution. Although there are no published studies which directly compare the lipid content of heat fixed and snap frozen tissues, recent studies have highlighted the importance of careful pre-analytical sample handling of tissue for lipid analysis as many lipids are unstable, prone to oxidation and breakdown by tissue lipases [37,38]. In one such study, heat fixation of brain tissue successfully prevented the post-sampling breakdown of lipids and the release of free fatty acids through heat inactivation of lipases [37]. In this respect, heat fixation using the Denator T1 stabilizor is a valuable tool for the preservation of the spatial distribution of lipids in tissues providing a representative “snapshot” of an organisms living state.

## Conclusions

Heat fixation of 95°C for 30 seconds using the Denator T1 Stabilizor can be used for the inactivation of ssRNA(+) enveloped viruses and Gram negative bacteria and is compatible with MALDI-MSI for protein/peptide and lipid analysis. To our knowledge this is the first report of the ability of this treatment to inactivate microbes. Further studies will be focused on the effectiveness of this treatment for the inactivation of other pathogenic viruses and bacteria. This tissue stabilization method is simple and rapid, and will facilitate the use of infected tissue samples from animal or human studies for downstream proteomic, small molecule drug detection, and imaging mass spectrometry analysis.

## Methods

### Animal Experiments and tissue mimetic model

Female BALB/c or C57BL/6 mice obtained from Charles River Laboratory (Frederick, MD) were infected intra-nasally (i.n.) or sub-cutaneously (s.c) with the viral pathogen Venezuelan equine encephalitis virus (VEEV), or the bacterial pathogens, *Burkholderia thailandensis* or *Burkholderia mallei*. Four mice total were infected with the vaccine strain of VEEV (TC83, i.n., 10^6^ pfu) or the virulent strain (Trinidad donkey: s.c., 10^4^ pfu). Four mice were infected with *B.thailandensis* and 3 mice were infected with *B.mallei*. (i.n., 10^5^ CFU). Additional inactivation studies were conducted using a tissue mimetic model. Briefly, kidneys were harvested from uninfected control animals used in the above studies and injected with one of the following bacterial isolates: *E.coli* (Top10, PGSV3-GmR pAM17-KanR), *Klebsiella pneumonia*, *Acinetobacter baumani* or *Burkholderia thailandensis,* ex-vivo using a 1 ml tuberculin syringe loaded with a 100 μl suspension of 3 × 10^8^ CFU/ml in PBS. One kidney was used for each bacterial isolate and one was used as an uninfected control.

Research was conducted under an IACUC approved protocol in compliance with the Animal Welfare Act, PHS Policy, and other Federal statutes and regulations relating to animals and experiments involving animals. The facility where this research was conducted is accredited by the Association for Assessment and Accreditation of Laboratory Animal Care, and adheres to principles stated in the Guide for the Care and Use of Laboratory Animals, National Research Council, 2011.

### Heat fixation

After active infection was established (4–5 days), the primary target organs for each pathogen were harvested: brain and spleen from VEEV- infected mice and lung and spleen from *Burkholderia*- infected animals. Brain and spleen were removed aseptically from each of the 4 mice infected with the vaccine strain of VEEV. Two of the tissue sets (brain and spleen) were quickly divided in half. One half was heat fixed and the other half was left untreated. These tissues were used for viral titer determination. The brain and spleen from the other two animals were heat fixed or snap frozen whole and were used for MALDI-MSI studies (see below). All tissues recovered from the mice infected with the virulent strain of VEEV were sectioned in half and used to make lysates for viral titer determination.

Lung and spleen harvested from 4 mice infected with *B. thailandensis* were also divided in half, with one half heat fixed and the other untreated. Three paired tissue sets (heat-fixed and untreated) were used for CFU determination and the last pair was processed for MALDI-MSI. For the tissue mimetic samples, kidneys harvested from mice were injected with one of six different bacterial isolates and incubated at 37°C for 1 hour. The tissues were divided in half. One half was heat fixed and the other half was left untreated.

Heat fixation was performed using the T1 Stabilizor (Denator, Gothenburg, Sweden). Briefly, tissues were placed in the stabilizer card (Denator) according to the manufacturer’s guidelines and the instrument was programmed to a “custom” setting of 95°C for 30 seconds (based on the “fresh structure preserving” method) with a heater position of 90%. The temperature, upper heater position and stabilization time are settings that can be adjusted when selecting the “custom” method option in the instrument. Tissue weights ranged from 34-260 mg before heat fixation.

### Viable pathogen determination

Tissue samples were weighed and lysates made with a Precellys®24-Dual (Bertin Technologies, Rockville, MD USA) bead beater device in 500 μl of RPMI-1640 media with 10% fetal bovine serum (FBS) for virus infected tissue or water for bacterial infected tissue. For the determination of viable virus from brain and spleen tissue, lysates were used in a plaque assay using Vero-76 cells. Cells were plated in 6-well plates 24 to 48 hours prior to use in assay, and plates were carried into the BSL-3 lab. SeaKem ME Agarose (Lonza, Rockland, MD, USA) was prepared for overlays by mixing an appropriate amount of agarose with an appropriate amount of Basal Medium Eagle (BME) to achieve the desired concentration (i.e., 1 g of agarose mixed with 100 ml of BME to achieve 1% agarose concentration). Agarose was melted by microwave in BSL-3 prior to use. Samples were diluted in 1× MEM (minimal essential media), supplemented with 10% FBS (vol/vol), in a 10-fold dilution scheme according to the experimental design. The culture medium in the 6-well plate was then removed by swift decanting and various dilutions of the virus inocula were added to duplicate or replicate wells in volumes of 100 μl. Inoculum fluid was distributed by manual gentle rocking. The plates were incubated at 37°C with 5% CO for about 1 hour. Without removal of the inoculum or washing the virus off of the monolayer, a primary liquid overlay containing a 1:1 ratio of 2× BME medium (supplemented with 10% FBS) equilibrated to 42°C and 1% melted agarose was overlaid in 2 ml aliquots onto the inoculated monolayer and allowed to solidify. Plates were incubated for approximately 24 hours and stained with 2 ml of secondary overlay which consisted of 1:1 volume ratio of 2× BME media + 10% FBS: 1% agarose, and the combined volumes were supplemented with 5% neutral red. Plaques were counted and recorded 24 hours post-staining.

For determination of viable bacteria, lysates from lung, spleen and the kidney mimetic model were tested by plating 100 μl of serial dilutions on LB or blood agar in duplicate and incubating for 48 h at 37°C. Bacterial load was calculated as an average of the countable colonies seen in each dilution.

### MALDI-MSI-tissue preparation and matrix application

Parallel tissue samples (heat-fixed and untreated) from the mouse infection studies were embedded in OCT (optimal cutting temperature cryo-compound), (Sakura Finetek, Torrence, CA, USA) freezing medium, immersed in a pre-chilled isopentane bath cooled with dry ice to freeze, and then stored frozen at −80°C. Sections (10 μm) were made with a Leica cryostat (Leica CM1900 Leica Microsystems Inc. Buffalo Grove, IL, USA) and were placed on ITO coated slides (Bruker Daltonics, Billerica, MA, USA) for MALDI-MSI. Serial sections (5 μm) were hematoxylin and eosin (H&E) stained for morphological analysis. After sectioning, the MALDI-MSI slides were rinsed in 70% ethanol for 30 seconds, followed by 95% ethanol for 30 seconds and a brief water wash to remove the OCT compound. Finally, the slides were again rinsed in 70% ethanol and 95% ethanol to dehydrate the tissue. After 1 hour in a dessicator, slides were sprayed with sinapinic acid (SA: 10 mg/ml in 50% acetonitrile, 1% TFA) for protein detection or 2,5-dihydroxybenzoic acid (DHB: 30 mg/ml in 50% methanol, 1% TFA) for lipid detection, in an ImagePrep spraying device (Bruker Daltonics) using the manufacturer’s recommended method for SA or DHB application respectively. After matrix application, the slides were stored in a dessicator under vacuum for 1 hour before MALDI-MSI.

### MALDI-MSI-instrument settings and data visualization

MALDI-MSI was performed on an AutoFlex III (Bruker Daltonics) equipped with a SmartBeam™ laser (Nd: YAG, 355 nm). For protein detection the instrument was operated in linear positive–ion mode over a mass range of 2-20 kDa, with a raster width of 100 μm and a laser focal laser spot size of 20 μm. Five hundred laser shots were summed at each location in a random walk pattern that collects 100 shot increments at five random areas within each location. The extraction voltage was 19.94 kV, the acceleration voltage was 18.64 kV, and the lens voltage was 6.98 kV. Calibration was performed externally using protein standard mix 1(Bruker Daltonics). For lipid detection, the instrument was operated in reflector mode with negative polarity over a mass range of 100–1800 Da with a raster width of 100 μm and 20 μm laser spot size, summing 500 shots at each location with an acceleration potential of 20.97 kV. Spectra were calibrated internally using the DHB matrix peaks of m/z 153.02: [M-H]^−^, and m/z 307.04: [2M-H]^−^. All MALDI mass spectrometry data was processed and images were viewed using Flex Imaging 4.0 (Bruker Daltonics). Peaks were selected with a mass window 0.4% for proteins and 0.1% for lipids and minimum intensity of 1%. All data was normalized to total ion current.

## Abbreviations

MALDI-MSI: Matrix assisted laser desorption ionization mass spectrometry imaging
VEEV: Venezuelan equine encephalitis virus
PFU: Plaque forming unit
CFU: Colony forming unit
OCT: Optimum cutting temperature
ITO: Indium tin oxide
BSL-3,4: Biosafety laboratory level 3 or 4
